# Pregnancy after bariatric surgery: a narrative literature review and discussion of impact on pregnancy management and outcome

**DOI:** 10.1186/s12884-018-2124-3

**Published:** 2018-12-27

**Authors:** Veronica Falcone, Tina Stopp, Michael Feichtinger, Herbert Kiss, Wolfgang Eppel, Peter Wolf Husslein, Gerhard Prager, Christian S. Göbl

**Affiliations:** 1 Department of Obstetrics and Gynecology, Division of Obstetrics and Feto-maternal Medicine, Medical University of Vienna, Währinger Gürtel 18-20, 1090 Vienna, Austria; 2 Wunschbaby Institut Feichtinger, Lainzerstrasse 6, Vienna, Austria; 3 Department of General Surgery, Division of Bariatric Surgery, Medical University of Vienna, Währinger Gürtel 18-20, 1090 Vienna, Austria

**Keywords:** Bariatric surgery, Pregnancy outcome, Pregnancy management, Narrative review

## Abstract

Bariatric surgery (BS) is regarded to be the most effective treatment of obesity with long lasting beneficial effects including weight loss and improvement of metabolic disorders. A considerable number of women undergoing BS are at childbearing age.

Although the surgery mediated weight loss has a positive effect on pregnancy outcome, the procedures might be associated with adverse outcomes as well, for example micronutrient deficiencies, iron or B12 deficiency anemia, dumping syndrome, surgical complications such as internal hernias, and small for gestational age (SGA) offspring, possibly due to maternal undernutrition. Also, there is no international consensus concerning the ideal time to conception after BS. Hence, the present narrative review intents to summarize the available literature concerning the most common challenges which arise before and during pregnancy after BS, such as fertility related considerations, vitamin and nutritional deficiencies and their adequate compensation through supplementation, altered glucose metabolism and its implications for gestational diabetes screening, the symptoms and treatment of dumping syndrome, surgical complications and the impact of BS on pregnancy outcome. The impact of different bariatric procedures on pregnancy and fetal outcome will also be discussed, as well as general considerations concerning the monitoring and management of pregnancies after BS.

Whereas BS leads to the mitigation of many obesity-related pregnancy complications, such as gestational diabetes mellitus (GDM), pregnancy induced hypertension and fetal macrosomia; those procedures pose new risks which might lead to adverse outcomes for mothers and offspring, for example nutritional deficiencies, anemia, altered maternal glucose metabolism and small for gestational age children.

## Background

There is a dramatic increase in overweight and obesity worldwide. The WHO estimates that 39% of adults worldwide are overweight (BMI ≥ 25 kg/m^2^) and 13% are obese (BMI ≥30 kg/m^2^) [1]. It is widely known that obesity is associated with numerous comorbidities such as hypertension, musculoskeletal disorders, cancer and type 2 diabetes [1–3]. Likewise, overweight or obese pregnant women show an increased risk for gestational diabetes [4], preeclampsia [5], spontaneous miscarriage [6], large for gestational age offspring and even fetal (neurological and cardiovascular) malformations [7]. Children from obese mothers may also develop health complications in later life, such as hypertension, diabetes or cardiovascular disease, due to epigenetic changes [8].

Weight loss is associated with improved fertility rates and pregnancy outcomes, [9], with BS having proven to be the most effective treatment [10]. However, BS itself can be a risk factor for the development of adverse pregnancy outcomes and poses a challenge for obstetricians. In the following narrative review, we will give a comprehensive overview on the benefits and risks of BS on pregnancy outcomes, like risk of malnutrition, maternal anemia, development of internal hernia, reduced risk of gestational hypertension and GDM (Table 1) and higher risk of SGA outcomes (Table 2).

## Overview of bariatric procedures

### Surgical techniques

Bariatric surgery might be indicated if other attempts of losing weight have failed. It is the most effective way for weight loss and the reduction of comorbidities like type II diabetes mellitus [11] and hypertension [12] and has favourable effects on cardiac function [13, 14]. International guidelines stipulate that BS should be considered if a patient’s BMI exceeds 40 kg/m^2^, or in case of a BMI between 35 kg/m^2^ and 40 kg/m^2^ with associated severe comorbidities; in the case of coexisting diabetes mellitus even in the case of a BMI between 30 kg/m^2^ and 35 kg/m^2^ [3].

BS is divided into restrictive and malabsorptive procedures or a combination of both. The most widely used surgical procedures are the Roux-en-Y gastric bypass (RYGB), the sleeve gastrectomy and the adjustable gastric band. Other techniques such as the biliopancreatic diversion are not very common and will therefore not be discussed in this review. RYGB (Fig. 1a) is a combined malabsorptive and restrictive procedure which consists of a horizontal partitioning of the upper part of the stomach to create a gastric pouch. 75 to 150 cm of the small intestine are used to create the alimentary limb which carries ingested food to the bowel without the addiction of biliopancreatic secretions which are carried directly into the bowel through the biliopancreatic limb, typically 30 to 60 cm in length [15].

Sleeve gastrectomy (Fig. 1b) is a restrictive procedure and is performed as laparoscopic gastric resection which creates a small gastric pouch. It can be combined with the duodeno-ileostomy as part of a biliopancreatic bypass [15].

Adjustable gastric banding (Fig. 1c) is normally performed as a laparoscopic procedure (LAGB) and consists in placing a band 1 to 2 cm below the gastroesophageal junction, creating an upper gastric pouch with a capacity of 20 to 30 mL. The degree of constriction of the stomach can be adjusted by the introduction of saline through the port [15].

Other less frequent procedures, such as the biliopancreatic diversion, will not be discussed in this paper.

### Less invasive endoscopic techniques

By the endoscopic placement of intragastric balloons with a volume of at least 400 ml gastric space is occupied and gastric motility is altered [16]. Compared to standard bariatric surgery, bariatric endoscopy (BE) is considered to be less invasive, more economic and associated with lower morbidity and mortality. Depending on individual circumstances, it might also be approved for patients with a BMI between 30 and 35 kg/m^2^ [16, 17]. Furthermore, the procedure can be repeated if necessary [17]. Bariatric endoscopy is associated with beneficial metabolic effects like reduced incidence of hyperuricemia, hypertriglyceridemia, hypercholesterolemia and diabetes mellitus [18]. To our knowledge there is currently only one retrospective study that investigated if BE has potential benefits for patients with obesity-induced infertility. The authors showed that 15 out of 27 obese women conceived successfully after the placement of an intragastric balloon and subsequent weight loss. All pregnancies were uneventful and ended with life births; however, further research is needed before concluding that BE is safe in reproductive age and pregnancy [19].

### Literature searching algorithm

The references for this review were obtained from Pubmed and MedLine databases using the MeSH Terms: “obesity”, “bariatric surgery”, “pregnancy and bariatric surgery”, “obesity and fertility”, “obesity and pharmacology”, “obesity and bariatric surgery”, “obesity and diabetes”, “diabetes and pregnancy”, “gestational diabetes and hypertension”, “obesity and hypertension”, “bariatric surgery and hypertension”, “obesity and heart disease”, “bariatric surgery and heart disease”, “gastric bypass and anaemia”, “gastric bypass and hyperparathyroidism”, “bariatric surgery and vitamin D”, “dietary supplements and gastric bypass”, “gastric bypass and abdominal hernia”, “fetal macrosomia”, “infant, small for gestational age, breastfeeding and bariatric surgery”. We prioritized longitudinal observational studies, cohort studies and meta-analysis. Furthermore, we used clinical guidelines from the American Congress of Obstetricians and Gynaecologists (ACOG) for the management of pregnancy and delivery after bariatric surgery and the Scientific Impact Paper on the role of bariatric surgery in improving reproductive health by the Royal College of Obstetricians and Gynaecologists.

## Challenges and benefits of Bariatric Surgery before pregnancy

Although obesity has become a major health care problem within the last years along with increasing prevalence of BS in women of childbearing age, there is no international consensus about management of pregnancy after BS. Even though BS seems to reduce obesity-related fertility issues and adverse pregnancy outcomes [20–22], obstetricians have to consider pregnancy related complications possibly caused by BS [20, 23].

The standard recommendation of the ACOG for women wishing to conceive after BS is to delay pregnancy for at least 1 to 1.5 years after surgery [23], which is also supported by the obesity management task force of the European Association for the Study of Obesity [24]. During the first post-surgical year a rapid weight loss is to be expected and becoming pregnant in this catabolic time frame could possibly lead to an altered nutritional supply to the growing fetus [21]. However, in contrast to these guidelines one recent study found no evidence supporting this recommendation [25].

Pregnancies after BS, especially malabsorptive procedures, are characterized by nutritional deficiencies such as anemia, low protein and vitamin levels [26, 27]. Furthermore, a history of BS is associated with altered glucose metabolism, impacting the diagnosis of hyperglycemia [28]. Recent data also indicates a higher risk for SGA offspring [22, 29] and one study found a statistically not significant trend towards higher rates of stillbirth or neonatal death [29]. In addition, pregnant women with a history of gastric bypass might be at risk to develop an internal hernia, potentially leading to severe consequences like bowel necrosis or acute perforation, which might eventually lead to acute C-Section [30]. Exceptional cases of maternal and fetal death have also been described [31, 32]. These aspects will be addressed and discussed in detail in the following paragraphs.

### Reproductive aspects

Obesity was shown to impact fertility on various levels by affecting endometrial and ovarian function [33–35]. Insulin resistance and compensatory hyperinsulinemia adversely affect intraovarian follicle growth and oocyte maturation, leading to oligo-/amenorrhea, hyperandrogenemia and polycystic ovarian syndrome (PCOS) [36, 37]. Consequently, a close interaction of impaired reproductive and metabolic features can be observed in obese women [38]. Hence, even at a young age, assisted reproductive technology (ART) is often required in obese patients to achieve a live-birth. The accompanying technical procedures such as ovarian ultrasound visualization or oocyte retrieval might be complicated by excess body weight [35]. Even when ART can be performed, obesity was associated with impaired treatment outcome including less collected oocytes after ovarian hyperstimulation, lower embryo quality, reduced pregnancy and live-birth rates and high miscarriage rates. Although the available data is still inconclusive, it seems that those impaired ART outcomes are attributable to obesity and not to underlying pathologies such as PCOS [39]. Therefore, in accordance with the general BS guidelines [3] and depending on the individual patient’s metabolic and reproductive profile, BS might be considered in infertile anovulatory patients with a BMI > 35 kg/m^2^ and no effect of life-style intervention for at least 6 months [40]. Bariatric surgery was shown to ameliorate hyperandrogenemia and PCOS in a majority of patients [41]. In patients trying to conceive after BS, one meta-analysis reported up to 58% spontaneous conception rates [42]. Moreover, self-esteem and sexual functioning are increasing following BS induced weight-loss [43]. Even patients undergoing ART before and after BS showed increased numbers of retrieved oocytes, improved oocyte quality and live-birth rates [44]. However, risks and benefits of BS at childbearing age should be carefully balanced, in order to improve maternal health and to reduce the risk of long-lasting health consequences in the offspring [35]. BS should not be regarded as a primary infertility treatment [23].

### Nutritional aspects

#### Deficiency Anemia

During pregnancy, hemoglobin (Hb) and hematocrit (Hct) levels decrease physiologically due to an expansion of blood volume by approximately 50% and red blood cell mass by only approximately 25% [45]. Pregnant women need to mobilize additional iron to meet the requirements of the growing fetoplacental unit, amounting to 1,200 mg during the course of pregnancy [46]. Although the absorption of iron is increased during pregnancy, it seems that an appropriate diet alone is not sufficient to meet those requirements, especially for women with a low pre-pregnancy iron status (Ferritin level < 30 μg/L) [47]. Thus, iron-deficiency anemia (IDA) is the most frequent form of anemia in pregnant women. According to the WHO, anemia, defined as Hb levels of < 11 mg/dl in pregnant women, affects 41.8% of this population subgroup worldwide, with iron deficiency accounting for approximately 50% of cases [48].

There also seems to be a link between obesity and an altered iron metabolism. Obesity is considered to be a state of chronic inflammation, leading to increased levels of the acute-phase reactant hepcidin which inhibits the enterocyte iron absorption. Other factors such as inflammatory-induced sequestration of iron to the reticuloendothelial system and higher iron requirements due to larger blood volume add to the association between obesity and hypoferremia [49].

Weight loss after BS results in falling serum hepcidin levels and potentially improved iron status [50]. Patients who underwent malabsorptive surgery, however, showed an increase in anemia rates (anemia prevalence from 12.2% at baseline to 25.9% after 2 years, prevalence of low ferritin levels from 7.9% at baseline to 23.0% after 2 years) which can be attributed to a reduced caloric intake, intolerance for red meat, reduced acid production of the stomach and subsequently decreased bioavailability of dietary iron and the bypass of food through the duodenum [26]. A history of BS before pregnancy seems to increase the risk for the development of IDA during pregnancy [51, 52]. One study indicates that the rate of severe anemia might be higher in pregnancies that occur more than 4 years after RYGB surgery, leading to the conclusion that the time to conception might also be of importance [53]. However, all studies on the topic have limitations and further research is required to reinforce the currently available supplementation recommendations for the prevention of IDA in pregnant women after BS [22, 52, 54]. As IDA during pregnancy has adverse effects on pregnancy outcome (e.g. an increased risk for preterm delivery [55, 56]), prevention is however crucial. Also, maternal iron deficiency seems to have long term health effects on the offspring, mainly neurobehavioral abnormalities and an elevated cardiovascular disease risk [46, 57]. The ACOG recommends a daily intake of 27 mg of ferrous iron during pregnancy for patients without a history of BS [45], the WHO recommends 30 to 60 mg of elemental iron [58]. According to the current literature, the recommended supplementation dose for the prevention of IDA in non-pregnant women with a history of BS is 45 to 130 mg iron daily [59, 60], whereas the currently available recommendation for pregnant women after RYGB ranges from 40 to 600 mg of ferrous iron daily [24, 61, 62]. Any dose within this range should therefore be applicable; however, frequent laboratory tests should be performed and the dose adapted according to the results [61, 62]. The ACOG recommends a complete blood count and measurement of iron and ferritin every trimester [23].

Folic acid and Vitamin B12 deficiency can also lead to maternal anemia. The folic acid demand increases from 50 to 400 μg per day during pregnancy and cannot always be met by diet alone, leading to folic acid deficiency being the most common cause for macrocytic anemia (MCV > 100 fL) during pregnancy [45]. Folic acid deficiency seems to be rare after all BS procedures [26, 63, 64]. The Endocrine Society Clinical Practice Guideline recommends biochemical monitoring preoperatively and 6, 12, 18 and 24 months after surgery and then in annual intervals only for patients after malabsorptive or combined procedures. A daily supplement of 400 μg of folic acid should also be performed [59]. The American Association of Clinical Endocrinologists also recommends pre- and postoperative routine screening only for patients after malabsorptive or combined BS and also a daily supplement of 400 μg of folic acid for all women of reproductive age [60]. Gascoin et al. compared non-obese pregnant controls with pregnant women after gastric bypass who took 800 μg/day of folic acid and did not observe folic acid deficiency in the bariatric group [63]. Weng et al. could also find no evidence of folate deficiencies in patients after RYGB. They suggest that folate absorption occurs throughout the entire small intestine and any deficiency caused by inadequate dietary intake can therefore easily be corrected by supplementation [26]. Jans et al. report folate deficiency in 0 to 16% of pregnancies after BS with no adverse clinical outcomes [54]. As there is still controversy regarding the benefit of folic acid supplementation on pregnancy outcomes [65], it seems prudent to follow the general folic supplementation recommendations for pregnant women and screen for folate deficiency every trimester [60]; which is also supported by the ACOG [23].

Vitamin B12 deficiency anemia is mostly seen in women after gastric resection or with Crohn’s disease [45]. The additional requirement of vitamin B12 during pregnancy is estimated to be 0.2 μg/day [66]. Vitamin B12 deficiency seems to occur especially after malabsorptive or combined BS as the secretion of intrinsic factor and gastric acid is decreased and the duodenum, being the main absorption site, is bypassed. Incidence of Vitamin B12 deficiency after RYGB is reported to be between 4 and 62% [59, 67], with a tendency to increase over the course of time, possibly due to the fact that the body’s reserves are able to cover the decreased absorption at early stages [26]. In pregnant women after BS, the prevalence of Vitamin B12 deficiency is reported to be between 48 and 53% [54], but not in bariatric gravidas who received a Vitamin B12 supplementation of 4 μg/day and 1,000 μg/month [63]. The Endocrine Society recommends biochemical monitoring preoperatively; 6, 12, 18 and 24 months after surgery and then in annual intervals only for patients after malabsorptive or combined procedures. With regards to the supplementation dose, recommendations for non-pregnant individuals range from 1,000 μg intramuscularly (im) every 3 months to 1,000 μg/week intranasally [59]. The American Association of Clinical Endocrinologists recommends pre-operative and annual screening for Vitamin B12 deficiency in patients after malabsorptive and combined bariatric procedures and a supplementation of 1,000 μg/day orally or 500 μg/week intranasally or 1,000 μg/month parenterally [60]. For pregnant women after BS Kaska et al. recommend 350 μg/day sublingually or 1,000 μg/month im [61] and Busetto et al. recommend 350 to 500 μg/day orally or 1,000 μg/month im or 3,000 μg every 6 months im or 500 μg/week intranasally [24]. Although the available data is still conflicting, vitamin B12 deficiency seems to be associated with a higher risk of preterm birth [68], recurrent abortion, low birth weight (LBW), intrauterine growth retardation (IUGR), neural tube defects and impaired cognitive development [69]. Therefore, obstetricians should assess the Vitamin B12 status of pregnant women after BS every trimester and treat deficiencies accordingly [24, 60].

#### Vitamin D, calcium and bone metabolism

Several studies have examined the relationship between post-BS pregnancy, calcium and vitamin D metabolism and found a Vitamin D deficiency in 3% to over 70% of pregnant women, depending on the BS procedure [51, 54, 70]. There is a physiological increase in the need of vitamin D and calcitriol during pregnancy seemingly related to the calcium transfer to the fetus, particularly in the last trimester [70].

Vitamin D is converted from 7-dehydrocholesterol by the skin after exposure to sunlight or provided through diet (oily fish, mushrooms, fortified cereals, egg yolks and dietary supplements). The ingested or converted vitamin D has to be activated in order to exert its functions, like increasing intestinal calcium uptake and promoting calcium and phosphate mobilization from the bone [71, 72]. The altered anatomy of the intestinal tract occurring especially after RYGB could directly interfere with calcium absorption, possibly leading to maternal bone loss, reduced calcium levels in breast milk or deficient fetal bone mineralization [61]. A possible association between vitamin D insufficiency during pregnancy and SGA offspring, perhaps by the impediment of intestinal calcium absorption or increase of inflammatory cytokines and cellular oxidative stress, is currently discussed [73–75].

Additionally, low vitamin D levels are often associated with higher levels of parathormone, causing secondary hyperparathyroidism and increasing the risk of accelerated bone remodeling, leading potentially, among other factors, to a lower bone mineral density in bariatric patients compared to non-surgical controls [76].

Inadequate Vitamin D levels (< 29 ng/ml) were observed in over 70% of pregnant women who underwent RYGB surgery, through all three trimesters of pregnancy and despite a supplementation with 600 IU of Vitamin D per day. The prevalence of elevated PTH levels (> 65 pg/ml) was highest in the third trimester with 32.6% of subjects. However, no adverse pregnancy outcomes were detected [70]. A large retrospective study conducted in Taiwan pointed out that there is a high incidence of post-surgery secondary hyperparathyroidism for all procedures (37.2%) which could lead to a higher long-term fracture risk, however, the available data ins still controversial. Long term follow up of the bone’s health in patients with a history of BS should however be considered [77]. Nutritional assessment, periodical blood examinations and aimed vitamin D supplementation are pivotal in maintaining physiological levels of vitamin D, calcium and PTH [24, 73, 74, 78]. The current US daily consumption recommendation for vitamin D is 600 IU and the toxicity limit is estimated to be between 10,000 and 40,000 IU/day [79]. The supplement dosage recommendations for post bariatric pregnant women range from 1,000 IU / day to 6,000 IU / day, with 1,000 to 2,000 mg of calcium citrate per day [24, 61]. Pregnant women should be screened for Vitamin D inadequacy at least once every trimester [23].

#### Protein deficiency

BS might be associated with protein deficiency as a consequence of the restricted food intake and absorption. Protein deficiency should be suspected in case of fatigue, weakness and hair loss. [80]. It can be diagnosed through clinical examination including muscle mass tests or, in case of severe protein deficiency, low serum albumin values [27, 80]. Patients occasionally develop edema and in rare cases anasarca [81, 82].

A German study in non-pregnant patients after BS provided evidence that 60 g/daily or even higher levels of protein supplements increase body fat mass loss without negative effects on the renal function [83].

The recommended protein intake for pregnant women after BS is 60 g daily [24] and the ACOG guidelines support this recommendation [23]. There is only little evidence for detrimental effects of maternal protein deficiency on pregnancy outcome, mainly impaired fetal growth [84]; however, pregnant women after BS should be advised to adhere to the general recommendations for post-surgery protein intake and the fetal growth should be assessed regularly [23, 24].

#### Other nutrients

The American Guidelines for the perioperative support of BS patients recommend routine screening for vitamin deficiencies, in order to prevent long term complications. For pregnant women, a screening every trimester is recommended [60].

Vitamin A deficiency was reported in 10% to 58% of pregnant women after BS, depending on BS procedure and gestational age [51, 61, 85].Vitamin A, alone or in combination with other fat-soluble vitamins (D, E, K), has to be supplied if deficiencies are present [60, 61]. Next to being an important antioxidant in the body, Vitamin A is also involved in cell signaling pathways. There is some evidence that antenatal Vitamin A supplementation reduces the risk of maternal anemia and the risk of maternal night blindness. Furthermore there is only weak evidence that antenatal vitamin A supplementation could reduce the risk of maternal infection [86]. The vitamin A supplement dose should not exceed 5,000 IU/day due to its teratogenic effects and should be administered in the form of beta-carotene [24, 61, 87].

Gascoin et al. observed also vitamin E deficiency in pregnant women with a history of gastric bypass, but no adverse pregnancy outcome are described [63].

Next to selenium, which plays an important role in several enzymatic reactions in the body, deficiencies of Vitamins C, B1 and B9 in pregnant women after BS were observed. Moreover, the offspring of mothers with a BS history displayed lower cord blood levels of several micronutrients such as Vitamin A, calcium, zinc and iron, in contrast to a control group [63].

Because of the limited number of participants in the available studies, no practical guidelines containing thresholds or dosage recommendations for the treatment of micronutrients deficiencies in post-surgical pregnancies have been created so far [51], however, all available statement papers recommend the supplementation of vitamins in pregnant women after BS [23, 24, 35, 61].

### Glucose metabolism and gestational diabetes

Gestational Diabetes Mellitus (GDM) is defined as “diabetes first diagnosed in the second or third trimester of pregnancy that is not clearly either preexisting type 1 or type 2 diabetes” [88] and affects approximately 6% of pregnancies in Europe [89]. Most recent guidelines recommend universal testing for GDM between 24 + 0 and 28 + 6 weeks of gestation by a 2 h 75 g oral glucose tolerance test (OGTT) [88]. The International Association of Diabetes in Pregnancy Study Group (IADPSG) established the following diagnostic thresholds: fasting plasma glucose ≥5.1 mmol/l (92 mg/dl), or 1-h plasma glucose ≥10.0 mmol/l (180 mg/dl), or 2-h plasma glucose ≥8.5 mmol/l (153 mg/dl) [90]. However, the diagnosis of GDM still remains controversial, as other diagnostic algorithms and thresholds are still in use [91], leading to heterogeneity in study results and epidemiologic data [89].

Obesity is a risk factor for the development of GDM. Compared to normal weight women, the OR for GDM was found to be 1.97 in overweight women (pre-pregnancy BMI 25 to 30), 3.01 in moderately obese (BMI 30 to 35) and 5.55 in severely obese women (BMI > 35) [92]. The mechanisms which link obesity and GDM are still a target of research, but the enhanced secretion of pro-inflammatory cytokines by adipose tissue and subsequent systemic inflammatory and immune dysregulation seems to increase the maternal insulin resistance [93, 94].

GDM is associated with a number of adverse pregnancy outcomes, especially cesarean section, large for gestational age, macrosomia and preeclampsia [91, 95]. Moreover, children of diabetic mothers seem to have an increased risk of developing obesity and metabolic dysfunction later in life [8, 96, 97] due to “metabolic imprinting”, e.g. the in-utero alteration of fetal organ function as a consequence of an excessive supply of nutrients and subsequently enhanced exposure to growth factors [96, 97].

BS before pregnancy seems to reduce the risk for developing GDM considerately [22, 52, 98–102]. Galazis et al. found the overall incidence of GDM as being approximately half in women after BS compared to controls [52]. However, results vary depending on control group and diagnostic criteria (see Table 1).

**Table 1:** Results of meta-analysis of studies comparing the risk for the development of GDM in women after BS with different subgroups, adapted from Galazis et al. [52]

Control Group	No. of Studies	Participants		OR (95% CI)	*p*-Value
		BS	Control		
Overall	15	2724	136,075	0.47 (0.40–0.56)	< 0.001
Women after BS vs. obese controls	6	1292	133,777	0.34 (0.18–0.67)	< 0.001
Women after BS vs. same women before BS	5	377	343	0.71 (0.45–1.11)	0.14
Women after BS vs. other women before BS	3	1171	916	0.42 (0.22–0,79)	0.007
Women after BS vs. pre-pregnancy BMI matched obese women without BS	3	433	1537	0.77 (0.22–2.65)	0.68
Women after BS vs. pre-surgery BMI matched obese women without BS	6	864	133,388	0.24 (0.10–0.54)	< 0.001

Despite the protective effect of BS and subsequent weight loss on the development of GDM, some procedures like RYGB alter glucose kinetics and might also have detrimental effects on pregnancy outcome and GDM diagnostics which have to be observed by obstetricians.

As previously observed in non-pregnant patients, some bariatric procedures (like RYGB and sleeve gastrectomy) are characterized by an exaggerated postprandial rise of plasma glucose concentrations followed by hyperinsulinemic hypoglycemia [103]. To provide first insights into the possible effects of gastric bypass surgery on glucose metabolism during pregnancy, we retrospectively assessed maternal characteristics of 76 pregnant women after gastric bypass. The data included results of a 2 h 75 g OGTT with measurements at fasting as well after 60 and 120 min after oral glucose load. We found that women after gastric bypass had improved fasting glucose, but altered patterns of postprandial glucose dynamics including a rise at 60 min, followed by hypoglycemia at 120 min in more than half of pregnant patients [28]. Our results were recently confirmed by another prospective cohort study on 25 pregnant women after RYGB, indicating that the recommended diagnosis criteria for GDM are not reliable after BS [104]. Obstetricians should consider other diagnostic approaches such as frequent capillary blood glucose measurements or continuous subcutaneous glucose monitoring (CGMS) in these patients; however, there are no guidelines yet [23, 24, 35, 105]. Only one study reported CGMS profiles of 35 pregnant women after RYGB and reported abnormal glucose variability in real-life conditions as well [106]. Therefore, obstetricians should be aware of symptoms indicative of dumping syndrome. The early dumping syndrome occurs within 15 min to 1 h after a meal rich in simple carbohydrates. The rapid emptying of hyperosmolar carbohydrates into the small intestine leads to a fluid shift from plasma to bowel, causing a drop in blood pressure and subsequent compensation, leading to vasomotor symptoms such as flushing, palpitation, perspiration, tachycardia, hypotension and syncope [107, 108]. Patients should be advised to consume smaller meals rich in complex carbohydrates, to delay liquid intake until at least 30 min after a meal and to lie down after eating to delay the gastric emptying into the small intestine [108]. The late dumping syndrome, with an onset of symptoms 2 to 3 h after a meal, is supposed to be caused by an excessive insulin response following the rapid glucose transit into the jejunum and subsequent reactive hypoglycemia [107, 108]. The symptoms include sweating, tremulousness, poor concentration, altered consciousness, palpitations and syncope. The main therapeutic intervention is a dietary modification eliminating refined carbohydrates. Pectin or guar gum can be added to increase viscosity of food but are poorly accepted due to their unpalatability. Diaxozide decreases the insulin release and has been reported to ameliorate the condition but is not safe in pregnancy; somatostatin analogues and acarbose are not well tested in pregnant human individuals and there is only one case report on successful treatment of late dumping syndrome with acarbose in a pregnant woman [107]. Obstetricians should seek advice from bariatric specialists if their pregnant patients present with symptoms indicative of dumping syndrome.

### Preeclampsia and hypertensive disorders

Hypertensive disorders in pregnancy include pre-gestational chronic hypertension, pregnancy-induced hypertension (PIH) and preeclampsia (PE). PE is defined as de novo onset of hypertension (> 140 mmHg systolic or > 90 mmHg diastolic) after 20 weeks gestation and the coexistence of at least one of the following conditions: proteinuria, other maternal organ dysfunction such as renal insufficiency, liver involvement or neurological complications or utero-placental dysfunction (fetal growth retardation) [109]. Hypertensive disorders affect approximately 10% [110] of all pregnancies and account for 14% of maternal deaths worldwide [111]. Its incidence is on the rise, with a 21% increase in inpatient deliveries involving PE between 2005 and 2014 in the USA [112]. Several authors attribute the increasing PE incidence to the obesity pandemic [113–115]. Mbah et al. report a positive association between PE incidence and pre-pregnancy BMI as well as pregnancy weight gain rate, with 3.3% of normal weight mothers being affected, 7.7% of mothers with class I obesity, 9.5% of mothers with class II obesity, 10.9% of mothers with class III obesity and 13.4% of super obese gravidas (BMI **≥** 50 kg/m^2^). In comparison to normal weight mothers, obese women had a three-fold increased risk for the development of PE [113]. Although the mechanisms by which obesity increases the risk for hypertensive disorders are not fully understood yet, it seems that obesity-related metabolic factors cause cytotrophoblast dysfunction and subsequent placental ischemia, thereby increasing the release of soluble placental factors and enhancing the sensitivity by which those factors cause endothelial dysfunction and hypertension [115]. With BS being the most effective treatment for obesity, it can be assumed that women who conceive after BS have a lower risk for developing hypertension disorders and the available data support this presumption. One study compared women who delivered before an already planned BS with women who delivered after BS. Almost 15% of women who delivered before BS had PE compared to only 3% of those who delivered after BS. Rates of PIH were also lower in the post-surgery group (2.5% versus 13.0%), resulting in a 75% lower odds to be diagnosed with a hypertensive disorder for women after surgery [116]. Several reviews and meta-analysis [22, 98–102, 117] come to the same conclusion. Yi et al. [102] report an overall OR of 0.42 for the diagnosis of hypertensive disorders in pregnancies after BS, with a significantly less OR (0.14) when conception took place within the first 2 years after surgery. Vrebosch et al. [99] come to the conclusion that the incidence of PE and PIH are lower in post-surgical women compared to obese non-surgical controls, but still higher than in normal weight women without BS**,** but only reviewed laparoscopic adjustable gastric banding studies. Ducarme et al. [118] found evidence that PE rates were lower in women after BS, but not different for PIH. Although the available data indicates that gravidas after BS are at significantly lower risk for the diagnosis of hypertension disorders, further research is needed, especially concerning the impact of different surgical procedures and surgery-conception time.

### Surgical complications

Pregnancy may expose women after BS to a higher risk of developing internal herniation due to the fact that the enlarged uterus lifts up the bowel, resulting in increased intra-abdominal pressure [24, 30]. In the case of acute abdominal pain, immediate surgical intervention must be considered, also when pregnancy has to be carried on [24, 30, 31, 119]. Of note, internal hernia after RYGB is not rare, with an incidence of up to 10% [30]. The most common internal hernias develop in the the transverse mesocolon defect, the Petersen’s space and the mesenteric defect underneath jejunu-jejunalis anastomosis [120]. Petersen’s hernia is a retroanastomotic hernia where the small bowel moves into the space between the caudal surface of the transverse mesocolon and the edge of the Roux limb and can rapidly lead to acute bowel obstruction with necrosis. In this case an immediate emergency surgery has to be performed [121]. Patients who are suspected to have developed an internal hernia are requested to fast during observation. If the abdominal pain relapses after the ingestion of food a subacute operation has to be considered. If the pain is constantly present in spite of fasting, an emergency operation (detorsion or bowel resection) is necessary and should be performed as fast as possible to minimize the risk of bowel necrosis and severe maternal and fetal complications [122].

### Fetal malformations

Obesity during pregnancy might be associated with a higher risk of fetal malformations like neurological defects, congenital heart defects and orofacial clefts. Furthermore, some data indicate that the risk of miscarriage and intrauterine fetal death could be increased [4]. A systematic review and meta-analysis assessed the risk of congenital anomalies in the offspring of obese pregnant women compared to lean pregnant women and found that neonates of obese women have a higher risk of neural tube defects (anencephaly OR: 1.39, CI: 1.03–1.87, spina bifida OR: 2.24, CI: 1.86–2.69), cardiovascular defects (OR: 1.30, CI: 1.12–1.51), and other congenital abnormalities such as anorectal atresia (OR 1.48, CI: 1.12–1.97), compared to pregnant women with normal BMI [7]. More recent studies come to similar conclusions [123].To date, the role of obesity in inducing fetal malformations is not fully understood and may reflect the difficulty of prenatal diagnosis at early pregnancy, due to obesity-related procedural difficulties. Further research is needed to elucidate the relationship between obesity and fetal malformations [123, 124].

### Fetal and neonatal complications

It is widely known that maternal obesity could lead to LGA offspring, which poses a high risk for complications during labour, like shoulder dystocia [125], and also to long-term health consequences, like obesity in childhood, diabetes and cardiovascular diseases [126]. Thus, it is reasonable to investigate if BS and consequent weight lost could also influence the children of mothers with a history of BS.

A Swedish national cohort study investigated the outcomes of 670 singleton pregnancies of post-surgical women and detected that pregnant women who underwent BS have a lower risk of gestational diabetes and large for gestational age (LGA) neonates, but a higher risk of SGA infants. No significant difference in the frequency of fetal malformations was found [29].

Several other studies (Table 2) found an increased risk of SGA infants born to mothers after malabsorptive or mixed bariatric surgeries [22, 52, 100, 102, 117], but not after solely restrictive procedures [52, 99]. The pathophysiology of this phenomenon requires further elucidation, but there seems to be an association between low maternal glucose levels in glucose challenge or oral glucose tolerance tests and SGA fetuses [95, 127]. An association between lower neonatal weight, glucose nadir and increased insulin release during an OGTT was most recently observed by our study group in offspring of mothers after RYGB [104]. In addition, Gascoin et al. found a significant inverse correlation between birth weight and length and maternal weight loss between surgery and pregnancy (the greater the weight loss the lower the birth weight and length). There were also low cord blood IGF1 and Leptin levels in infants from RYGB mothers, hinting to a decreased anabolism in those infants [63]. Low birth weight seems to have detrimental effects on the offspring even in adulthood. Being born SGA is considered to be a risk factor for the development of insulin resistance and type 2 diabetes, the metabolic syndrome and cardiovascular diseases [128], possibly due to fetal programming by changes in the intrauterine environment in malnourished mothers (thrifty phenotype hypothesis) [129]. Therefore, it might even be considered to prefer restrictive over malabsorptive BS techniques in young women who have a desire to bear children to avoid those complications [52].

**Table 2:** Overview on the SGA risk after bariatric surgery, comparing malabsorptive to restrictive surgery, adapted from Johansson [29] Gascoin [63] Chevrot [139] Sheiner [101] and Ducarme [118]

Control Group	Study	Participants	SGA	SGA after malabsorptive surgery	SGA after restrictive surgery	*p*-Value
Women after BS vs obese controls	Johansson	670/2356	15.6% vs 7.6%	n.d.	n.d.	< 0.001
Women after BS vs lean pregnant women	Gascoin	56/56		n.d	n.d.	NS
Women after malabsorptive surgery vs women with restrictive surgery	Chevrot	58/81	n.d.	17	7	< 0.001
Women after restrictive vs women after malabsorptive surgery	Sheiner	394/55	n.d.	7.3	12.8	NS
Women after RYGB vs women after LAGB	Ducarme	31/63	n.d.	32.3%	17.1%	NS

However, two retrospective studies conducted in Israel and in France compared fetal birth weight after malabsorptive and restrictive procedures and found no statistically significant difference in SGA rates between the two groups [130, 131].

### Breastfeeding

Human breast milk is a rich source of carbohydrates, protein, fat, vitamins, minerals, digestive enzymes and hormones (87% water, 3.8% fat, 1.0% protein, and 7% lactose). Additionally, it contains a vast amount of other, at least partially bioactive compounds, such as immune cells and human milk oligosaccharides (HMOs). These HMOs were found to exert antibacterial effects in the infant’s gastrointestinal tract. Regarding micro-nutrition, human milk supplies sufficient amounts of all vitamins except Vitamin D and vitamin K. Therefore, the lack of these two vitamins carries out some risk of deficiency for the infant [132].

Vitamin B12 deficiency might be a problem in breastfed infants born to women after gastric bypass, potentially leading to detrimental consequences such as polycythemia or megaloblastic anemia [133]. As observed in one case the milk secreted by lactating women after gastric bypass could be of lower nutritional density, especially in milk fats. This could lead to delayed growth of the children when breastfed exclusively as it was observed in one case report [134]. However, breastfeeding is known to prevent several infectious, atopic and cardiovascular diseases. Breastfeeding may also reduce the risk of respiratory infections, asthma, leukaemia and sudden infant death syndrome [135]. It also provides positive effects on brain and neuronal development and might be associated with a higher IQ [136]. Other studies concluded that exclusive breastfeeding for longer than six months may reduce the risk of obesity in later life [137]. As there is very little evidence regarding nutrient deficiencies in breast milk after BS, it is reasonable to recommend bariatric patients to breastfeed their infants [24, 138]. The above-mentioned positive effects of human breast milk most likely outweigh any BS related deficiency. However, there is no international consensus regarding vitamin or micronutrient supplementation during the lactational period after BS and healthcare professionals should take the patients history of BS into consideration when their infants present with symptoms of any nutritional deficiency.

### Limitations

The limitations of this study result from its narrative approach. Compared to systematic reviews or meta-analysis, narrative reviews are characterized by subjective study selection and weighing. Inclusion criteria and study characteristics are mostly unspecified which may cause misleading in drawing conclusions. To be able to elaborate objective guidelines for the management of pregnancies after BS, systematic reviews and meta-analysis should be performed.

## Conclusion

History of BS is associated with several risks for the mother and the fetus. Women who want to conceive should have a preconception counseling to be informed about the risks of pregnancy after BS, like malnutrition, deficiency and subsequent supplement of micronutrients, internal hernia and SGA infants. Regular blood examinations and regularly performed ultrasounds of the growing fetus (growing-curve, umbilical Doppler, amniotic fluid index) are necessary. Furthermore, the OGTT should not be performed as a routine test for the screening of gestational diabetes, because of the high risk of hypoglycemia. Ideally, pregnant women should be taken care of by a specialized center offering a multidisciplinary team with experience in the management of pregnancies after BS.

Any severe upper abdominal pain must be taken seriously because of the high risk of internal hernia. Of note, an international treatment consensus for pregnancy after BS is missing due to its novelty; hence specific recommendations of way of delivery or breastfeeding are not yet available. However, within the next years the number of pregnant BS patients and possible complications will increasingly challenge obstetricians.

## Abbreviations

ACOG: American Congress of Obstetricians and Gynaecologists
ART: Assisted Reproductive Technology
BE: Bariatric endoscopy
BMI: Body Mass Index
BS: Bariatric surgery
CGMS: Continuous subcutaneous glucose monitoring
C-section: Cesarean section
GDM: Gestational diabetes mellitus
Hb: Hemoglobin
Hct: Hematocrit
HMO: Human milk oligosaccharides
IADPSG: International Association of Diabetes in Pregnancy Study Groups
IDA: Iron deficiency anemia
IGF1: Insulin-like growth factor 1
im: intramuscular
IQ: Intelligence quotient
IU: International Units
IUGR: Intrauterine growth retardation
LAGB: Laparoscopic gastric banding
LBW: Low birth weight
LGA: Large for gestational age
MCV: Mean Corpuscular Volume
OGTT: Oral glucose tolerance test
OR: Odds ratio
PCOS: Polycystic Ovary Syndrome
PE: Preeclampsia
PIH: Pregnancy-induced hypertension
PTH: Parathyroid hormone
RYGB: Roux-en-Y gastric bypass
SGA: Small for gestational age
WHO: World Health Organization
